# Non-communicable diseases and preventive health behaviors: a comparison of Hispanics nationally and those living along the US-Mexico border

**DOI:** 10.1186/s12889-015-1850-y

**Published:** 2015-06-19

**Authors:** Belinda M. Reininger, Jing Wang, Susan P. Fisher-Hoch, Alycia Boutte, Kristina Vatcheva, Joseph B. McCormick

**Affiliations:** University of Texas Health Science Center – School of Public Health, Brownsville Regional Campus, One West University Blvd, RAHC, Brownsville, TX 78520 USA; University of Texas Health Science Center at Houston School of Nursing, 6901 Bertner, Houston, TX 77030 USA; University of Texas Health Science Center – School of Public Health, Austin Regional Campus, 1616 Guadalupe, Suite 6.300, Austin, TX 78701 USA

**Keywords:** Hispanics, Non-communicable diseases, Preventive health behaviors, US-Mexico border

## Abstract

**Background:**

Non-communicable diseases (NCDs) are rising among US Hispanics, but few studies have examined the preventive health behaviors for these NCDs among Hispanics. This study compared the preventive health behaviors of smoke-free living, physical activity, fruit and vegetable consumption, and avoidance of heavy alcohol use in Hispanics in the United States and Hispanics living along the US-Mexico border.

**Methods:**

Two weighted data sets with information on Hispanic populations were analyzed: 1) the national Behavioral Risk Factor Surveillance Survey (n = 29,942) from 2009; and 2) the Cameron County Hispanic Cohort (n = 1,439) recruited from the US-Mexico border between 2008–2011. To compare the preventive health behaviors of the samples, within a generalized estimating equation framework, weighted univariate and multivariate logistic regression analyses were conducted controlling for age, educational attainment, employment, language, and insurance status. Statistical tests were two-sided with a significance level set at 0.05.

**Results:**

Both samples reported low engagement in preventive behaviors. However, Hispanic males and females from the US-Mexico border were significantly less likely than the national sample to meet physical activity and fruit and vegetable consumption guidelines. Also, Hispanic males from the US-Mexico border were more likely to engage in heavy alcohol use.

**Conclusion:**

The lack of preventive health behaviors among Hispanics living along the US-Mexico border presents a dire prospect for NCD control in the region. Multipronged approaches to address multiple behaviors should be considered.

## Background

The burgeoning of non-communicable diseases (NCDs) worldwide brings a growing threat to public health and to economies: costs are projected to exceed $30 trillion over the next 20 years [1, 2]. NCDs now account for more than two-thirds of deaths globally [3], and in the United States (US), NCDs including cardiovascular disease, cancer, chronic respiratory disease and diabetes, contribute to 7 out of 10 deaths [1, 4, 5]. NCDs also disproportionately affect low-income and minority populations in the US [6]. They are pronounced among Mexican Americans, especially those living along the US-Mexico border, the geographical region extending north from Mexico into the US for 100 km [7]. Recent research shows that Hispanics living along the US-Mexico border experience levels of NCDs resembling the morbidity and mortality rates found in low-and middle-income countries [1, 8].

NCDs are among the most preventable of diseases, primarily because they are a result of individual health behaviors [4]. Preventive health behaviors, including smoke-free living, physical activity, fruit and vegetable consumption, and avoidance of heavy alcohol use, help to prevent or alleviate the suffering and early mortality related to NCDs [4] (see Table 1). It is estimated that if behavioral and environmental risks associated with NCDs were removed it is estimated that at least 80 % of all heart disease, stroke, and Type II diabetes and more than 40 % of cancer cases could be prevented [9]. However, little research examined behaviors preventive of NCDs among Hispanics, particularly along the US-Mexico border. This study therefore examined the extent to which preventive health behaviors were self-reported among Hispanics, primarily of Mexican descent, in two weighted samples, one from the US as a whole and the other from the US-Mexico border. Given the documented chronic disease prevalence along the border, we expected to see fewer preventive health behaviors, including smoke-free living, physical activity, fruit and vegetable consumption, and avoidance of heavy alcohol use in Hispanics living along the border than in Hispanics nationally.

**Table 1:** Guidelines and data on preventive health behaviors associated with non-communicable diseases (NCDS)

Behavior	Guidelines and Facts
Smoke-free Living	● The Surgeon General’s report indicates that there is no acceptable level of exposure to cigarette smoke. Cigarette use is the most preventable source of disability, disease, and death in the US [36].
	● Approximately 4 of 5 American adults are current non-smokers. However, about 43 million American adults currently smoke [4], and an estimated 443,000 people die prematurely each year from smoking or exposure to secondhand smoke [40].
	● Approximately 84 % of US Hispanic adults report not smoking, compared to 78 % of non-Hispanic whites [41].
Physical Activity	● The US Department of Health and Human Services (DHHS) recommends that adults complete 150 minutes of moderate-intensity activity or 75 minutes of vigorous-intensity activity per week [42].
	● Physical activity has numerous health benefits, particularly related to achieving and maintaining a healthy weight which reduces risks for NCDs [43].
	● Based on the DHHS 2008 Physical Activity Guidelines for Americans, less than two-third of all adults meet physical activity recommendations [42].
	● Hispanics nationally are less likely to be physically active than their non-Hispanic White counterparts [29, 31–33].
Fruit and Vegetable Consumption	● Men aged between 31–50 years should consume approximately 2 cups of fruit and 3 cups of vegetables per day [19].
	● Women aged between 31–50 years should consume approximately 1 ½ cups of fruit and 2 ½ cups of vegetables per day [20].
	● Following nutritional guidelines is associated with maintaining a healthy weight and reduces the risk for cardiovascular diseases, some cancers, and diabetes [4].
	● Despite over 20 years of promotion of fruit and vegetable consumption, in 2009 only 23.5 % of US adults consumed the daily recommended servings of fruits and vegetables [14].
	● Hispanics report higher levels of fruit consumption (37.2 %) than white non-Hispanics (31.1 %) but lower levels of vegetable consumption (19.7 % vs. 27.7 %) [44].
	● Multiple environmental and sociocultural factors appear to contribute to this disparity, including acculturation and the food environment of Hispanic communities [45, 46], with poor access to healthy foods and grocery stores but high access to fast food options [47].
Avoid Heavy Alcohol Use	● US Dietary Guidelines recommend that alcohol be consumed only in moderation, a maximum of two drinks for men and one drink for women per day [48].
	● One alcoholic drink is equal to 12 oz of beer, 5 oz of wine, or 1.5 oz of distilled spirits [49]. Moderate or excessive consumption of alcohol is associated with acute myocardial infarction [50], cancers of the mouth, esophagus, liver, colon, and breast [51], hypertension, alcoholic cardiomyopathy, and cerebrovascular events [52]. It is the nation’s third-leading lifestyle related cause of death [53].
	● Hispanics report lower rates of heavy drinking (6.1 %) than Native Americans (12.1 %) or whites (8.3 %), but Hispanic males report the highest levels of daily heavy drinking (40 %) of all groups (Native Americans 29.34 %, whites 30.74 %) [54].

## Methods

The study used two datasets containing detailed information on Hispanic populations. One sample included 29,942 Hispanic respondents from the national Behavioral Risk Factor Surveillance System (BRFSS) in 2009 [10]. The other sample included a total of 1,408 participants from the Cameron County Hispanic Cohort (CCHC) who were enrolled between the years 2008 and 2011 [11].

BRFSS is a national on-going telephone survey of self-reported health conducted yearly by the Centers for Disease Control and Prevention [10]. Data were collected over a 12 month period in 2009 for all states and territories of the United States [10]. For this study, the sample was restricted to respondents self-identified as Hispanic. Identification of Hispanic subgroups by country of ancestral descent is unavailable via BRFSS, but, 63 % of Hispanics nationwide identify as of Mexican descent [10].

The CCHC, a prospective cohort study, began in 2004 and includes adults aged 18 years and older living in Brownsville, TX, a low-economic midsize city along the US-Mexico border with a population that is 93.2 % Hispanic, with 86.2 % of Mexican descent [12]. Cohort participants were recruited from randomly selected households using a two-stage cluster systematic sampling method. Participants received clinical examinations, answered a behavioral health questionnaire, and had anthropometric measures taken; a description of these has been previously published elsewhere [11]. Surveys were conducted in the participant’s preferred language (English or Spanish) by trained bilingual staff. Data on tobacco use, physical activity, fruit and vegetable consumption, and alcohol use were drawn from participants’ behavioral measures. Participants of Mexican descent were identified by ancestry variables when a participant, parent, or grandparent indicated nativity in Mexico. The Committee for the Protection of Human Subjects at the University of Texas Health Science Center at Houston approved all protocols and consent forms.

### Smoke-free living

To assess the prevalence of smoke-free living, CCHC participants were asked, “Do you now smoke cigarettes?” with a response of “yes” labeled as smoker and “no” labeled as nonsmoker. BRFSS respondents were asked, “Do you now smoke cigarettes every day, some days, or not at all?” with “every day” and “some days” responses collapsed to indicate a smoker, and a response of “not as all” labeled as nonsmoker [15].

### Physical activity

To assess the extent of physical activity, CCHC participants were assessed using validated scales to examine moderate and vigorous physical activity of at least 10-minute bouts during the past 7 days [16, 17]. On the BRFSS, respondents reported the number of days per week and the total time per day of moderate and vigorous physical activity of at least 10 minute bouts [15]. CCHC and BRFSS survey items were used to estimate metabolic equivalents (METs) minutes of energy expenditure in the past 7 days, where participants with > 600 MET adjusted minutes were considered to meet physical activity guidelines.

### Fruit and vegetable consumption

Using the Two-item Food Frequency Questionnaire [18], fruit and vegetable consumption was assessed in the CCHC by asking participants how many portions of fruit and vegetables they ate daily. A portion size was described as a ½ cup of fresh, frozen, or canned produce or a medium-sized piece of produce. Consumption of five or more fruit and vegetable portions was considered meeting guidelines [19, 20]. On the BRFSS, participants reported the number of servings of fruits and vegetables daily, and those who reported five or more servings per day were considered to meet guidelines [15].

### Heavy alcohol use

CCHC participants were asked the number of alcoholic drinks (12 oz. beer, 8 oz. wine cooler, 4 oz. wine, or 1 oz. liquor) consumed each week. The total was divided by 7 to obtain an estimated daily number of alcoholic drinks consumed. In the BRFSS, one alcoholic drink was measured as a 12 oz. beer, a 5 oz. glass of wine, or one shot of liquor [15]. For both samples, heavy alcohol use for males was identified as having more than two drinks per day and for females having more than one drink per day [15].

### Sociodemographic variables

Data on age, gender, educational attainment (high school diploma or less than high school diploma) employment (employed or unemployed), preferred language (English or Spanish), and health insurance (insured or uninsured) were calculated on both samples.

### Statistical analysis

To derive representative population-based estimates, all analyses were performed using sampling weights for the CCHC and the BRFSS samples. The two samples were compared based on sociodemographic characteristics and preventive health behaviors using generalized estimating equations (GEE) correlated outcomes to account for potential clustering among participants within CCHC households and census tracts and blocks. Specifically, to compare proportions by dataset (CCHC or BRFSS) and obtain crude and adjusted p-values, we used univariate and multivariate GEE regression models, respectively, with logit link function for binomial responses. Similarly, to compare means by dataset and obtain crude and adjusted p-values, we used univariate and multivariate GEE regression models with identity link function for continuous responses. All statistical tests were two-sided with a significance level of 0.05. Analyses were performed using SAS version 9.1 [21].

## Results

On average, sampled participants from the CCHC were significantly older than BRFSS participants (CCHC: 47.22 ± 1.0 BRFSS: 40.47 ± 0.2, p-value < 0.0001) (Table 2). The CCHC sample also had a higher proportion of females than the BRFSS sample (CCHC: 58.3 %, BRFSS: 49.7 %, p-value < 0.0004), and a higher percentage of participants who did not graduate from high school (CCHC: 47.9 %, BRFSS: 31.2 %, p-value < 0.0001). Among CCHC participants, the percentage employed was significantly lower than among BRFSS participants (CCHC: 47.6 %, BRFSS: 55 %, p-value = 0.0041). When stratified by gender, there were fewer employed males (CCHC: 63.8 %, BRFSS: 65.6 %, p-value 0.6493) and fewer employed females (CCHC: 36.0 %, BRFSS: 44.3 %, p-value = 0.0017) in the CCHC sample.

**Table 2:** Sociodemographic characteristics of CCHC (2008–2011) and BRFSS (2009) Hispanic respondents

	CCHC	BRFSS	Difference
	n = 1408 n (%) or Mean ± SE	n = 29942 n (%) or Mean ± SE	(p value)
**All Participants**			
Mean Age	47.22 ± 1.0	40.47 ± 0.2	<0.0001
Gender			
Male	446 (41.7)	10817 (50.3)	0.0004
Female	961 (58.3)	19125 (49.7)	
Educational Attainment			
High School Diploma	635 (52.1)	20639 (68.8)	<0.0001
< High School Diploma	772 (47.9)	9064 (31.2)	
Employment			
Employed	680 (47.6)	14552 (55.0)	0.0041
Unemployed	727 (52.4)	15129 (45.0)	
Language			
English	295 (26.7)	15098 (52.6)	<0.0001
Spanish	1093 (73.3)	12362 (47.4)	
Insurance			
Insured	435 (38.5)	16907 (65.2)	<0.0001
Uninsured	961 (61.5)	7144 (34.8)	
**Males**			
Mean Age	45.3 ± 1.6	39.7 ± 0.3	0.0019
Less than H.S. graduate	213 (43.3)	3244 (31.5)	0.0049
Currently employed	299 (63.8)	6398 (65.6)	0.6493
Surveyed in Spanish	325 (67.3)	4394 (46.9)	<0.0001
Has insurance	162 (38.8)	6033 (63.6)	<0.0001
**Females**			
Mean Age	48.6 ± 1.3	41.2 ± 0.2	<0.0001
Less than H.S. graduate	559 (51.2)	5820 (31.0)	<0.0001
Currently employed	381 (36.0)	8154 (44.3)	0.0017
Surveyed in Spanish	768 (77.6)	7968 (47.8)	<0.0001
Has insurance	273 (38.3)	10874 (66.9)	<0.0001

CCHC, Cameron County Hispanic Cohort; BRFSS, Behavioral Risk Factor Surveillance System

Table 3 summarizes the preventive health behaviors of smoke-free living, meeting physical activity guidelines, fruit and vegetable consumption, and avoiding heavy alcohol use. After adjusting for age, educational attainment, employment, preferred language, and insurance, BRFSS respondents were significantly more likely than CCHC participants to meet recommended physical activity guidelines (44.14 % vs. 33.3 %, adjusted p-value = 0.0186). BRFSS respondents were also significantly more likely than CCHC participants to meet fruit and vegetable consumption guidelines (21.93 % vs. 14.8 %, adjusted p-value = < 0.0001).

**Table 3:** Preventive Health Behaviors of ncds among CCHC (2008–2011) and BRFSS (2009) Hispanics respondents

Preventive health behaviors	CCHC	BRFSS	Difference	Adjusted
	n = 1408 n (%)	n = 29942 n (%)	(p value)	(p-value^1^)
**Smoke-free Living**				
Smoker	186 (16.15)	4132 (14.72)	0.4493	0.3640
Non-smoker	1097 (83.9)	25383 (85.28)		
**Meet PA Guidelines**				
Yes	410 (33.3)	10982 (44.14)	<0.0001	0.0186
No	970 (66.7)	15797 (55.86)		
**Meet F&V Consumption Guidelines**				
Yes	198 (14.8)	6358 (21.93)	<0.0001	<0.0001
No	1024 (85.2)	21689 (78.07)		
**Avoid Heavy Alcohol Use**				
Heavy Use	50 (4.7)	884 (4.38)	0.6966	0.0656
No Heavy Use	1027 (95.3)	27538 (95.62)		

^1^Calculated adjusted p-values adjusting for age, educational attainment, employment, language, and insurance

NCDs, non-communicable diseases; CCHC, Cameron County Hispanic Cohort; BRFSS, Behavioral Risk Factor Surveillance System; PA, physical activity; F&V, fruits and vegetables

Table 4 summarizes preventive health behaviors stratified by gender for both samples. When adjusted for age, educational attainment, employment, preferred language, and insurance, BRFSS male respondents were significantly more likely than CCHC male participants to meet fruit and vegetable consumption guidelines (46.61 % vs. 39.8 %, adjusted p-value = 0.0034). BRFSS male respondents were also significantly more likely to avoid heavy alcohol use (93.86 % vs. 91.0 %, adjusted p-value = 0.0236). Among females, BRFSS respondents were significantly more likely to meet physical activity guidelines (41.68 % vs. 39.8 %, adjusted p-value = 0.0015) and to consume adequate fruits and vegetables (25.16 % vs. 11.4 %, adjusted p-value = 0.0417).

**Table 4:** Preventive Health Behaviors of NCDs among CCHC (2008–2011) and BRFSS Hispanics respondents (2009) by gender

	CCHC	BRFSS	Difference	Adjusted
	n = 1408 n (%)	n = 29942 n (%)	(p-value)	p-value
**Males**				
Smoke-free Living				
Smoker	109 (27.0)	1968 (19.69)	0.0191	0.1595
Non-smoker	306 (73.0)	8672 (80.31)		
Meet PA Guidelines				
Yes	155 (39.8)	4319 (46.61)	0.1097	0.8075
No	283 (60.2)	5333 (53.39)		
Meet F&V Consumption Guidelines				
Yes	42 (11.4)	1821 (18.71)	0.0256	0.0034
No	347 (88.6)	8262 (81.29)		
Avoid Heavy				
Alcohol Use				
Heavy Use	42 (9.0)	501 (6.14)	0.0907	0.0236
No Heavy Use	322 (91.0)	9581 (93.86)		
**Females**				
Smoke-free Living				
Smoker	77 (8.1)	2164 (9.68)	0.3624	0.8947
Non-smoker	791 (91.9)	16711 (90.32)		
Meet PA Guidelines				
Yes	155 (39.8)	6663 (41.68)	0.1097	0.0015
No	283 (60.2)	10464 (58.34)		
Meet F&V Consumption Guidelines				
Yes	42 (11.4)	4537 (25.16)	0.0256	0.0417
No	347 (88.6)	13427 (74.84)		
Avoid Heavy				
Alcohol Use				
Heavy Use	8 (1.6)	383 (2.63)	0.2703	0.7754
No Heavy Use	675 (98.4)	17957 (97.37)		

^1^Calculated adjusted p-values adjusting for age, educational attainment, employment, language, and insurance

NCDs, non-communicable diseases; CCHC, Cameron County Hispanic Cohort; BRFSS, Behavioral Risk Factor Surveillance System; PA, physical activity; F&V, fruits and vegetables

## Discussion

This study found Hispanics nationally and Hispanics living on the US-Mexico border reported low engagement in preventive health behaviors, and Hispanics living on the border engaged the least. These findings are important since Hispanics are the fastest growing population in the US and thus will influence future wellness projections and health care costs. Previous reports on disease rates from the US-Mexico border have shown significantly higher rates of overweight, obesity, and diabetes than in national samples [9, 10, 22, 23]. The results of this study suggest that the population along the US-Mexico border will continue to have higher levels of NCDs. The study found that Hispanics living on the US-Mexico border were significantly less likely to engage in the preventive health behaviors of physical activity and fruit and vegetable consumption than Hispanics nationally. Also Hispanic males nationally were significantly more likely than Hispanic males on the US-Mexico border to avoid heavy alcohol use.

The limitations of this study include differences in data collection and sampling between the two datasets. In-person interviews were used to collect behavioral information from participants in the CCHC, while BRFSS data were collected through telephone interviews. This difference introduced potentially different respondent – interviewer biases since they did not sit face-to-face during the BRFSS interviews as they did in the CCHC study. However, wording of questions was similar for both samples and coding for preventive behaviors of interest was standardized in the analysis.

It should also be noted that the majority of CCHC participants were of Mexican descent, while BRFSS respondents were probably more diverse in their representation of Latin-descent populations. However, while past research on preventive health behaviors has shown differences between Latin descent populations in alcohol use [24–26], tobacco use [27–30], and physical activity [31–33], there are larger differences between this group and other racial/ethnic groups [32–37]. A final limitation to this study is the possible under-sampling of those with severe disparities among BRFSS participants resulting from the intent of BRFSS to produce results generalizable to the entire US. When considering national surveys, it is thus important to pay attention to under-sampled, understudied, but highly disparate areas such as the US-Mexico border.

## Conclusions

Despite its limitations the study provides clear evidence of disparities in preventive health behaviors for NCDs among Hispanics along the US-Mexico border. The lack of preventive health behaviors among Hispanics along the border is particularly disturbing because it mirrors features found among low and middle income populations worldwide, where NCDs are escalating [3]. High rates of poverty among the rapidly growing population along the US-Mexico border, low levels of educational attainment, and lack of health insurance increase the complexity of addressing NCDs. Common obstacles faced in addressing disparities in preventative health behaviors include food insecurity, abundant access to unhealthy foods, and insufficient environmental infrastructure to promote preventive behaviors [38]. Our findings suggest that health resources should be used to disseminate and implement evidence-based practices, programs, and policies to promote preventive behaviors. A multipronged approach would span the ecological model [39] such that preventive health behaviors could be aligned with system and policy changes to support individuals in adopting and maintaining such behaviors long-term. Our findings also suggest a need for synergistic approaches to nurturing multiple preventive behaviors, rather than addressing each one as an isolated behavior. Given the disparities found in NCDs faced by Hispanics nationally and on the US Mexico border, particularly, multipronged approaches to foster preventive health behaviors are overdue.
